# Effect of crop type shift on soil C:N:P stoichiometry in a typical Yellow River irrigated district

**DOI:** 10.1038/s41598-025-16151-w

**Published:** 2025-08-17

**Authors:** Bo Bo, Rong-bo Zhao, Yang Wang, Chun-hua Li, Zi-jian Xie, Chun Ye

**Affiliations:** 1 National Engineering Laboratory for Lake Pollution Control and Ecological Restoration, Chinese Research Academy of Environmental Sciences, Beijing, China; 2 Key Laboratory for Lake Pollution Control of the Ministry of Ecology and Environment, Chinese Research Academy of Environmental Sciences, Beijing, China

**Keywords:** Yellow River irrigation area, Stoichiometric ratio, Soil degradation, Crop type change, Ecology, Environmental sciences

## Abstract

The Hetao irrigation area is one of the largest irrigation areas in the Yellow River Basin and is a typical salinized agricultural area. Crop type shifts can affect soil stoichiometric ratio characteristics, indicating soil nitrogen and phosphorus availability and the soil degradation process. However, few studies have been conducted in this area. In this study, soil samples were collected at 45 sites under sunflower fields (SF) and other land (OL), and the SF were mostly shifted by the OL. The results revealed that the soil pH and salinity clearly increased while the OL shifted to the SF. Moreover, soil organic matter (SOM), total nitrogen (TN), and total phosphorus (TP) also decreased significantly. These findings indicate that the soil degradation process accelerated during crop type conversion. Moreover, with the transition from OL to SF, there was little difference in the C: N ratio, whereas the C:P and N:P ratios decreased significantly. The soil P mineralization rates increased, and the N limits improved during the crop type shift. In addition, the soil C:N, C:P, and N:P ratios decreased overall with increasing soil depth. This research provides new insight for the management of crop types and the improvement of soil properties in the saline‒alkali soil of the Yellow River irrigation area.

## Introduction

Eco-chemometrics is a science that integrates the basic principles of physics, chemistry, biology and other disciplines to study the balance and interaction of chemical elements in ecological processes^1–3^. Soil carbon (C), nitrogen (N) and phosphorus (P) are essential elements for plant growth and have important impacts on the soil microbial community structure, organic matter decomposition, plant growth and other processes, affecting the response and feedback of ecosystems to global change^4^. The soil C:N:P stoichiometric ratio can not only indicate the soil C, N and P cycles and their coupling relationships but also reflect the heterogeneity of the soil ecosystem structure and function^5^. For example, the C: N ratio has an important influence on the soil microbial activity and mineralization rate, which in turn affects the soil carbon and nitrogen cycles, whereas the C: P ratio affects soil P fixation and mineralization^6^. Thus, the study of the soil C: N:P stoichiometric ratio is an effective method for revealing the regulation and feedback of environmental changes in the soil element composition.

Arable land is the most commonly utilized land. Owing to changes in crop types, water and fertilizer management, the quantity and properties of fertilizers, animal and plant residues, and microorganisms in the soil have changed, resulting in differences in the migration and transformation processes of soil C, N, and P^2^. Chen et al.^7^ reported that soil C increases when farmland is converted to forest or grassland. Moreover, the conversion of farmland to shrub land or wild grassland is more beneficial for C retention than the conversion of farmland to artificial forest. Li et al.^8^ found that the C: N:P ratios of woodland and upland soil is basically equal, which is lower than that of paddy soil, mainly due to the differences in altitude, vegetation types and field management. Additionally, the C: N and C: P ratios clearly increased with decreasing nitrogen and phosphorus application rates. Therefore, it is useful to analyse soil C: N:P stoichiometric ratios to reveal soil nutrient circulation and redistribution patterns as land use changes.

The Hetao Irrigation District in Inner Mongolia is one of the main grain-producing areas in the Yellow River Basin of China, with a high degree of soil salinization^9^. The area of salinized cultivated land has reached 3.94 × 10^5^ hm^2^, accounting for 68.7% of the total cultivated land in this area^10^. Sunflower fields (SF), corn fields (CF), wheat lands (WL) and vegetable and fruit lands (VFL) are the major crop types, accounting for more than 95% of the total cultivated area. As soil salinization has increased, the regional crop type structure has obviously changed, with numerous other lands (OL) shifting to SF^11^. The stoichiometric ratio is an important indicator reflecting soil properties, which are strongly affected by changes in fertilization and irrigation methods caused by crop type shifts^12^. However, few studies have investigated the effects of crop type variation on the soil C: N:P stoichiometric ratio and its variation across different soil depths in the Hetao irrigation area.

The aims of this study were (i) to investigate the soil physicochemical properties and C: N:P ratios under the effects of crop type changes and soil depth and (ii) to clarify the major driving factors affecting the soil C: N, C: P, and N: P ratios.

## Materials and methods

### Study area

The study area is located in Wuyuan County, Bayannur City of the Inner Mongolia Autonomous Region, China. The regional climate is mid-temperate continental climate, with an annual average temperature and rainfall of 3.7℃–7.6℃ and 130–285 mm, respectively; the annual average evaporation is 2030–3180 mm^13^. The main source of river water is agricultural irrigation canals and drainage canals. The main irrigation periods are spring irrigation (April–May), summer irrigation (June–September) and autumn irrigation (October–November).

Wuyuan County is an important grain-producing area in Bayannur City. According to the Statistical Yearbook of Bayannur City, the area under crop cultivation in Bayannur City in 2023 was 758,727 hm^2^, whereas in Wuyuan County, the area was 143,567 hm^2^, accounting for almost 1/5 (2023) of the total area (http://tjj.bynr.gov.cn/, accessed on 20 October 2024). From 2011 to 2023, the crop planting area in Wuyuan County increased by 12,113 hm^2^, with a growth rate of 9.2%. WL, CF, SF, and VFL were the main crop fields, accounting for 2, 29, 56, and 5%, respectively, of the cultivated land area in Wuyuan County (96%). The SF area has increased rapidly from 38 to 56% over the past twelve years, with an average ratio of 49%. Conversely, OL decreased significantly from 62 to 44%.

### Sample collection and analysis

**Fig. 1:**
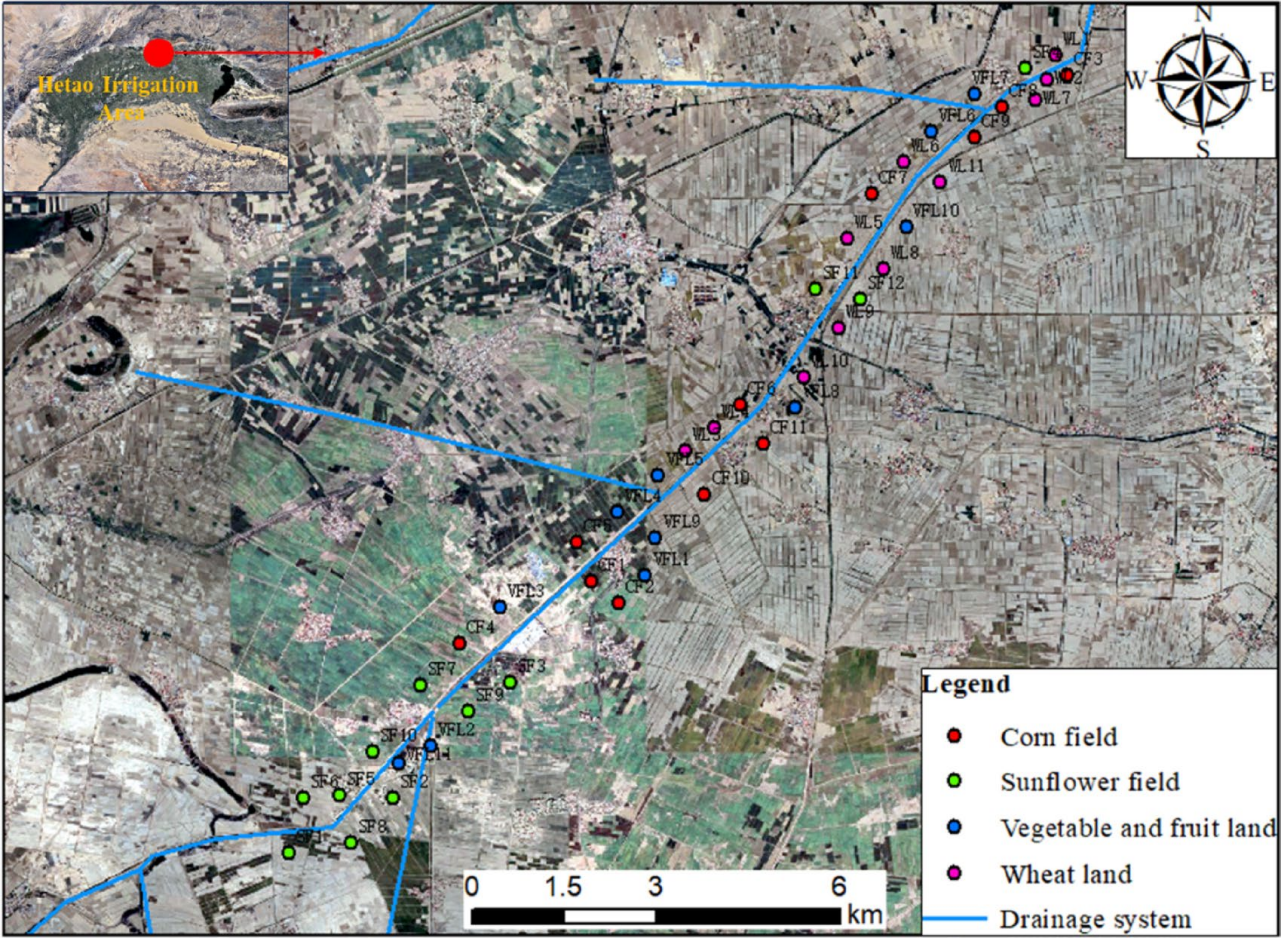
Locations and the sampling sites in the study area.

The sampling area is located in the sixth drain (Fig. 1, made with ArcGIS 10.2; data from the geospatial data cloud: www.gscloud.cn/, accessed on 8 October 2024). A total of 225 soil samples were collected from 45 sampling sites (12 in SF and 33 in OL, including WL, CF, and VFL) at five soil depth profiles (0–20 cm, 20–40 cm, 40–60 cm, 60–80 cm, 80–100 cm) in September 2023. Before sample collection, the soil surface litter was removed clearly and penetrated a soil drill to 100 cm deep into the ground to collect five soil profiles samples evenly. SF were mostly shifted by OL, with crop changes occurring over a period of 5–10 years. The collected samples were naturally air-dried until they reached a constant weight. After drying, the samples were ground, sieved, and then divided into fractions using 10-mesh, 60-mesh and 100-mesh sieves. Among them, the samples that had passed through a 10-mesh sieve were used to measure the soil pH and soil salinity. The samples that have passed through a 60-mesh sieve were used to determine the soil organic carbon (SOC), while the samples that have passed through a 100-mesh sieve were used to determine the total nitrogen (TN) and the total phosphorus (TP)^14,15^.

The soil pH was measured with a pH meter (S8 meter) at a 1:2.5 soil: water ratio (HJ 962–2018). The soil salinity was determined by the gravimetric method (DB37T 1303–2009). The SOC content was determined by hydrated hot potassium dichromate oxidation-colorimetry^14^. Soil organic matter (SOM) was obtained by converting the measured SOC values using a coefficient conversion method^14^. Soil particle size was measured by a laser particle size analyser (Mastersizer 2000). The TN content was determined by the selenium powder‒copper sulfate‒potassium sulfate‒concentrated sulfuric acid digestion‒semimicro Kjeldahl method^15^. The TP was digested in a HNO_3_‒HF microwave and determined by ICP‒OES (Optima 5300DV).

### Data analysis

IBM SPSS Statistics 27, Origin 2022 and Canoco 5.0 were used to analyse and draw the data. One-way ANOVA and the least significant difference (LSD) test were performed using IBM SPSS Statistics 27 (IBM Statistical Package for the Social Sciences Statistics, Version 27, https://www.ibm.com/cn-zh) to determine the differences in soil CNP levels and their stoichiometry among different crop types. Statistical analyses, including Pearson correlation and linear regression, were conducted using OriginPro 2022 (OriginPro 2022, Version 9.9, https://www.originlab.com) to evaluate the effects of various environmental factors on soil C: N:P stoichiometry. Redundancy analysis (RDA) was performed using Canoco 5.0 software (Canonical Community Ordination, Version 5.0, https://www.canoco5.com/index.php) to visualize the relationships between the soil physicochemical properties and the soil C: N:P stoichiometry.

## Results and discussion

### Effects of crop type changes on basic soil physical and chemical properties

Generally, the soil was weakly alkaline across the entire soil depth profile. The pH value in SF was 8.8 at the soil depth of 0–20 cm, which was significantly greater than that in the OL (8.4) (*P* < 0.05) (Table 1). As the soil depth increased, the pH in the OL gradually increased, with an increase of 0.2 units. In SF, the pH initially increased but subsequently decreased, with the highest value occurring in the 40–60 cm layer. Importantly, the differences in pH reached a significant level in each layer (*P* < 0.05) (Table 1).

**Table 1:** Soil physical and chemical properties of different crop types.

Depth	Crop types	pH	Salinity	SOM	Sand	Silt	Clay
		(−)	(g/kg)		(%)		
0–20	SF†	8.8 ± 0.5**a**‡	2.8 ± 4.4a	8.6 ± 1.8**b**	62.2 ± 12.5a	32.0 ± 9.4a	5.8 ± 3.7a
	OL	8.4 ± 0.2**b**	1.8 ± 2.6a	12.3 ± 3.9**a**	66.1 ± 10.4a	29.2 ± 9.2a	4.8 ± 2.3a
20–40	SF	9.1 ± 0.6**a**	2.3 ± 1.2a	10.0 ± 4.7a	69.0 ± 15.5a	26.8 ± 12.7a	4.3 ± 3.5a
	OL	8.5 ± 0.1**b**	0.9 ± 0.6a	9.4 ± 3.1a	60.7 ± 24.5a	28.0 ± 11.1a	4.6 ± 2.3a
40–60	SF	9.2 ± 0.6**a**	1.8 ± 1.0a	7.0 ± 1.8a	64.7 ± 14.8a	29.0 ± 10.3a	6.3 ± 4.6a
	OL	8.5 ± 0.2**b**	1.0 ± 0.6a	6.6 ± 2.1a	66.8 ± 26.2a	22.9 ± 10.9a	2.9 ± 2.1a
60–80	SF	9.1 ± 0.6**a**	1.5 ± 1.0a	6.8 ± 2.5a	59.1 ± 16.0a	33.4 ± 11.9a	7.4 ± 4.2a
	OL	8.6 ± 0.3**b**	1.4 ± 0.8a	8.0 ± 1.5a	54.7 ± 28.4a	33.9 ± 17.9a	5.3 ± 4.4a
80–100	SF	9.0 ± 0.5**a**	2.3 ± 1.6a	7.3 ± 2.0a	55.0 ± 16.5a	35.9 ± 13.8a	9.1 ± 3.9a
	OL	8.6 ± 0.3**b**	1.3 ± 0.9a	7.1 ± 2.6a	57.4 ± 26.2a	30.4 ± 15.1a	5.7 ± 3.3a

^†^OL, other land, including WL, VFL and CF.

^‡^All values are presented as the means ± SD. Significant differences between SF and OL are denoted by different lowercase letters (*P* < 0.05).

The salinity in the SF was greater than that in the OL in each layer (Table 1). Salinity values are highest in the 0–20 cm layer, with values of 2.8 and 1.8 g/kg for SF and OL, respectively. With increasing soil depth, the salinity initially decreased but then increased, with the lowest value occurring in the 40–60 cm layer.

With respect to the SOM, the value in SF was 8.6 g/kg in the 0–20 cm layer, which was significantly lower than that in OL (12.3 g/kg) (*P* < 0.05) (Table 1). SOM level in SF and OL was classified as Grade IV and V, respectively, and are deficient in organic matter (Nutrient grading standard of the second national soil survey, 1979) (Table 2)^16^. The difference was not significant below the 0–20 cm layer (*P >* 0.05). The soil was classified as sandy loam. The particle composition was mainly sand, accounting for 54.7–69.0%, followed by silt and clay, accounting for 22.9–35.9% and 2.9–9.1%, respectively (Table 1). There was no significant difference for the entire soil depth profile between SF and OL (*P >* 0.05). However, the sand content decreased at soil depths of 60–80 cm and 80–100 cm, whereas the silt and clay contents slightly increased.

**Table 2:** The nutrient classification standard of the second National soil survey (g/kg)^17^.

Indicator	Ⅰ-extremely rich	Ⅱ-rich	Ⅲ-relatively rich	Ⅳ-moderate	Ⅴ-poor	Ⅵ-extremely poor
SOM	>40	30 ~ 40	20 ~ 30	10 ~ 20	6 ~ 10	< 6
TN	>2	1.5 ~ 2	1 ~ 1.5	0.75 ~ 1	0.5 ~ 0.75	< 0.5
TP	>1	0.8 ~ 1	0.6 ~ 0.8	0.4 ~ 0.6	0.2 ~ 0.4	< 0.2

SF exhibited significantly better growth performance than did OL under saline-alkaline soil conditions. As a result, more farmland has been converted to planted sunflowers as the soil salinity has increased. Sunflowers have a well-developed root system that continuously absorbs soil salt as they grow, allowing them to grow in high-pH and high-salinity environments^18,19^. In contrast, OL have the lowest pH and salinity, indicating that they are strongly affected by the intensification of salinization. Thus, nearly 16% of the cultivated area shifted from OL to SF from 2011 to 2023. SOM in the OL was greater than that in the SF. pH was negatively correlated with SOM (*P <* 0.05), indicating that the soil is becoming less fertile as the soil is becoming more saline. Crop type had no significant influence on the soil particle composition, whereas the silt and clay contents increased in the deep layer soil. Concentrated irrigation increases the leaching of silt and clay particles, which leads to increases in the silt and clay contents at soil depths ranging from 60 to 100 cm^20–22^.

### Effects of crop type changes on SOC, TN and TP contents

The TN content in the SF was 0.70 g/kg at the soil depth of 0–20 cm, which was significantly lower than that in the OL (1.00 g/kg) (*P* < 0.05) (Table 3). TN levels in SF and OL soil were classified as Grades Ⅲ and V, respectively, indicating moderate and deficient TN levels (Nutrient grading standard of the second national soil survey, 1979) (Table 2)^16^. The TN in the 0–20 cm layer of the OL soil was greater than that in the other layers of the soil, whereas for the SF soil, the highest value occurred at a soil depth of 20–40 cm.

The TP concentration was 909.3 mg/kg in the OL, which was higher than that in the SF (848.0 mg/kg) (Table 3). TP levels in SF and OL soil were classified as Grade II, indicating a rich TP level (Nutrient grading standard of the second national soil survey, 1979) (Table 2). The TP in the OL was greater than that in the SF across the entire soil depth profile and tended to decrease with increasing soil depth (Table 3). The SOC contents in the SF and OL ranged from 3.96 to 5.80 and 3.81–7.13 g/kg, respectively. The changes in SOC correlated with that in SOM (Tables 1 and 3).

**Table 3:** SOC, TN and TP in different crop types.

Depth	Crop types	SOC	TN	TP
		(g/kg)		(mg/kg)
0–20	SF†	5.00 ± 1.03**b**‡	0.70 ± 0.17**b**	848.0 ± 229.1a
	OL	7.13 ± 2.23**a**	1.00 ± 0.27**a**	909.3 ± 150.0a
20–40	SF	5.80 ± 2.74a	0.80 ± 0.26a	705.0 ± 299.1a
	OL	5.47 ± 1.77a	0.78 ± 0.17a	776.8 ± 68.1a
40–60	SF	4.03 ± 1.04a	0.58 ± 0.09a	664.0 ± 102.1a
	OL	3.81 ± 1.22a	0.61 ± 0.09a	730.0 ± 45.9a
60–80	SF	3.96 ± 1.46a	0.64 ± 0.15a	637.0 ± 78.9a
	OL	4.66 ± 0.87a	0.75 ± 0.11a	730.6 ± 37.7a
80–100	SF	4.21 ± 1.15a	0.56 ± 0.14a	609.3 ± 121.3a
	OL	4.14 ± 1.50a	0.58 ± 0.11a	688.6 ± 80.3a

^†^OL, other land, including WL, VFL and CF.

^‡^All values are presented as the means ± SD. Significant differences between SF and OL are denoted by different lowercase letters (*P* < 0.05).

SOC, TN and TP were derived primarily from the application of chemical fertilizer and organic fertilizer and from the mineralization of SOM and parent materials^9,20^. The SOC, TN and TP contents in the SF were lower than those in the OL, and the differences reached a significant level at the soil depth of 0–20 cm (*P <* 0.05) (Table 3). On the one hand, fertilizer application was the main reason for this difference. The organic fertilizer, nitrogen fertilizer, and phosphate fertilizer applied in the SF were 4557, 268, and 168 kg/hm^2^, while those applied in the OL ranged from 6852 to 8418, 265–418, and 244–267 kg/hm^2^. On the other hand, sunflowers are grown in areas with high soil pH, salinity, and low SOM content, which is not conducive to fertilizer conservation^23,24^. Moreover, the SOM and TN contents in SF and OL plots both decreased with increasing soil depth (Table 3). Concentrated irrigation increases the leaching of N and P, which leads to an increase below the ploughing layer^25,26^. With respect to the irrigation season, the groundwater depth in the study area can reach < 100 cm, which could result in the loss of N and P nutrients in the deep layer.

### Effects of crop type changes on the soil C:N:P stoichiometric ratio

The soil C: N ratios in the SF and OL soils ranged from 6.12 to 7.24 and from 6.18 to 7.17, respectively (Table 4). The C: N ratio in the SF soil was greater than that in the OL soil at the soil depth of 0–60 cm but lower than that in the OL soil at 60–100 cm. As the soil depth increased, the C: N ratio first decreased, followed by a slight increase in the 80–100 cm layer. The soil C: P ratios in the SF and OL soils ranged from 6.01 to 7.93 and from 5.21 to 7.87, respectively. The soil N: P ratios in the SF and OL ranged from 0.85 to 1.11 and 0.84–1.11, respectively. For the 0–20 cm layer, the C: P and N: P ratios were significantly greater in the OL than in the SF, whereas for the other layers, the difference did not reach a significant level (*P >* 0.05).

The soil C: N ratio is an important indicator for assessing the soil nutrient balance and can be used to measure the mineralization rate of SOM, thus evaluating the ability of soil to release nutrients^5^. Generally, the lower the C: N ratio is, the faster the soil mineralization rate, and the higher the rate of nutrient release^6^. Compared with the average level in China (11.9)^1^, the C: N ratio in the study area is lower, which means that the soil mineralization speed in the study area is greater and that it can quickly replenish the nutrients consumed and absorbed from the soil. Moreover, crop type shifts had no significant effect on the soil C: N ratio.

The soil C: P ratio can reflect the availability and mineralization ability of soil P. The higher the C: P ratio is, the lower the soil P availability and mineralization rate; thus, the lack of P limits the decomposition of microorganisms^1,5^. The C: P ratio in the study area is much lower than the average level of soil in China (61), suggesting enhanced crop utilization and mineralization rates in this region^27^. Additionally, the crop type shift from OL to SF significantly decreased the C: P ratio (*P <* 0.05), indicating that the soil mineralization speed increased as the crop structure shifted. The mineralization speed was the main reason for the difference in SOC content. The higher SOC content in the plough layer contributed to the application of organic fertilizers and straw residue, whereas for the deep layer soil, the leaching of SOM particles increased the SOC content^7^. According to Table 1, the particle composition of silt and clay increased with increasing soil depth. Notably, the relatively high rate of phosphorus loss in deep soils is another reason for the relatively high C: P ratio.

Soil N and P serve as essential nutrients for plant growth, yet their availability can also limit growth by regulating key enzyme activities^28,29^. The N: P ratio can be used to judge the degree of soil N saturation and thus determine the limiting nutrients that are beneficial for plant growth^30^. Generally, a N: P ratio < 10 indicates that the soil is limited by N, a N: P ratio > 20 indicates that the soil is limited by P, and N: P ratios between 10 and 20 are limited by both elements^6^. The N: P ratio in the study area is much lower than the average level of soil in China (5.2), indicating that the soil is affected by N limitation^1^. In addition, a shift in crop type from OL to SF significantly decreased the N: P ratio (*P <* 0.05) and accelerated soil N limitation.

**Table 4:** C: N, C: P and N: P ratios for different crop types.

Depth	Crop types	C: N	C: P	N: P
		(−)		
0–20	SF†	7.24 ± 1.33a‡	6.10 ± 1.37**b**	0.85 ± 0.16**b**
	OL	7.17 ± 1.78a	7.84 ± 1.78**a**	1.11 ± 0.22**a**
20–40	SF	6.96 ± 1.50a	7.93 ± 2.81a	1.11 ± 0.20a
	OL	6.85 ± 0.95a	6.96 ± 1.82a	1.00 ± 1.57a
40–60	SF	6.89 ± 0.77a	6.01 ± 0.66a	0.88 ± 0.08a
	OL	6.18 ± 1.40a	5.21 ± 1.52a	0.84 ± 0.11a
60–80	SF	6.12 ± 0.88a	6.29 ± 2.46a	1.00 ± 0.25a
	OL	6.26 ± 0.96a	6.37 ± 1.11a	1.02 ± 0.15a
80–100	SF	6.77 ± 2.49a	7.12 ± 2.65a	0.91 ± 0.08a
	OL	7.00 ± 2.05a	6.00 ± 1.91a	0.86 ± 0.17a

^†^OL, other land, including WL, VFL and CF.

^‡^All values are presented as the means ± SD. Significant differences between SF and OL are denoted by different lowercase letters (*P <* 0.05).

### Correlation analysis between the soil C:N:P ratios and environmental factors

Crop type shift is the driving factor affecting the soil C: N:P ratio. The redundant analysis revealed that the contribution of crop types reached 23.5% and was the largest factor behind SOM and TN (Fig. 3). As the crop type shifted from OL to SF, the C: P ratio significantly decreased (*P <* 0.05, Table 4). The difference in fertilization caused by different crop types is the main reason for the difference in the C: N:P ratio. The organic fertilizer, nitrogen fertilizer, and phosphate fertilizer applied in the SF were 4557, 268, and 168 kg/hm^2^, respectively, whereas those applied in the OL ranged from 6852 to 8418, 265–418, and 244–267 kg/hm^2^, respectively. Moreover, differences in irrigation mode and root length are another important reason for changes in the C:N:P ratio^11,20^.

**Fig. 2:**
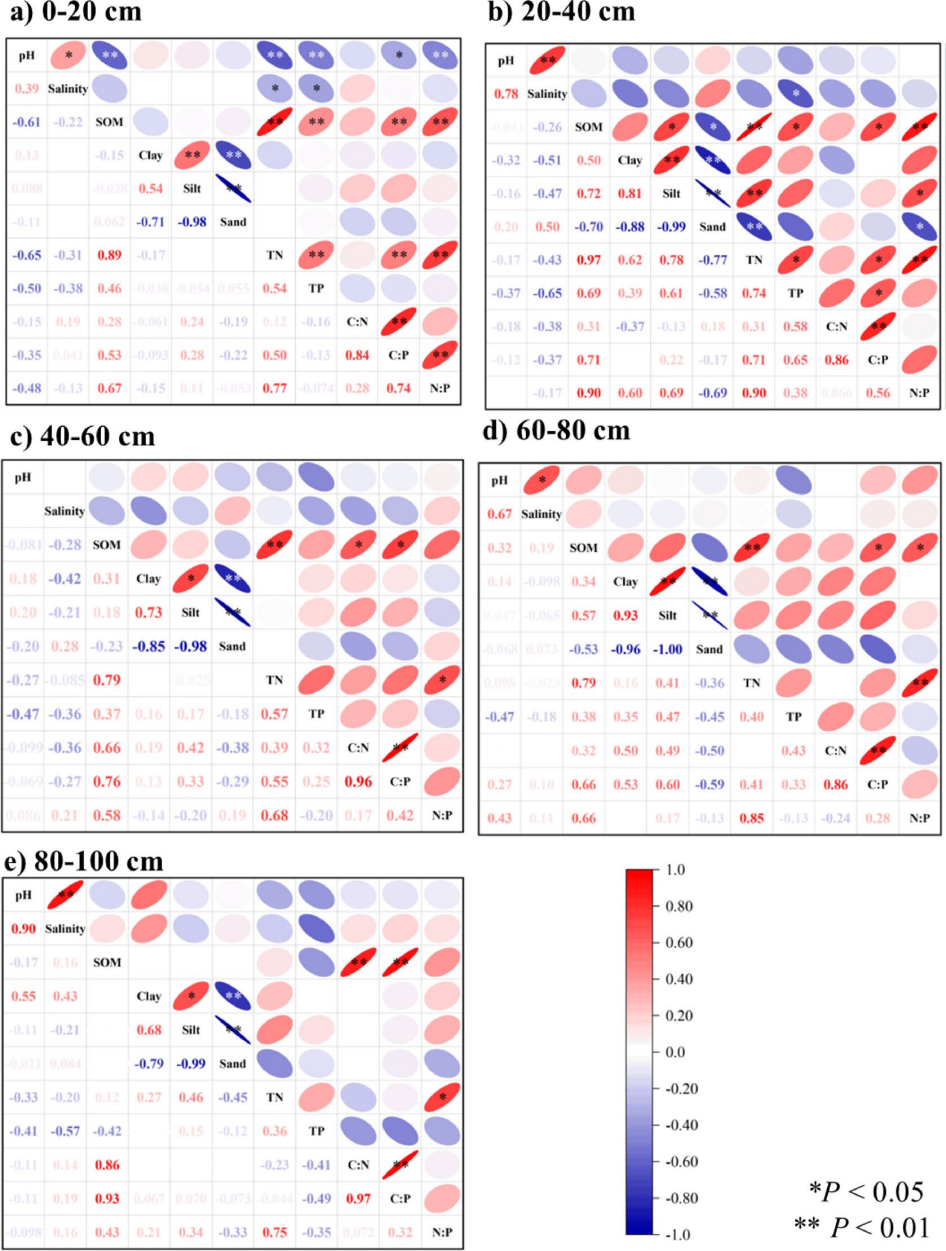
Correlation analysis of the soil C:N:P ratio and environmental factors at different soil depths.

**Fig. 3:**
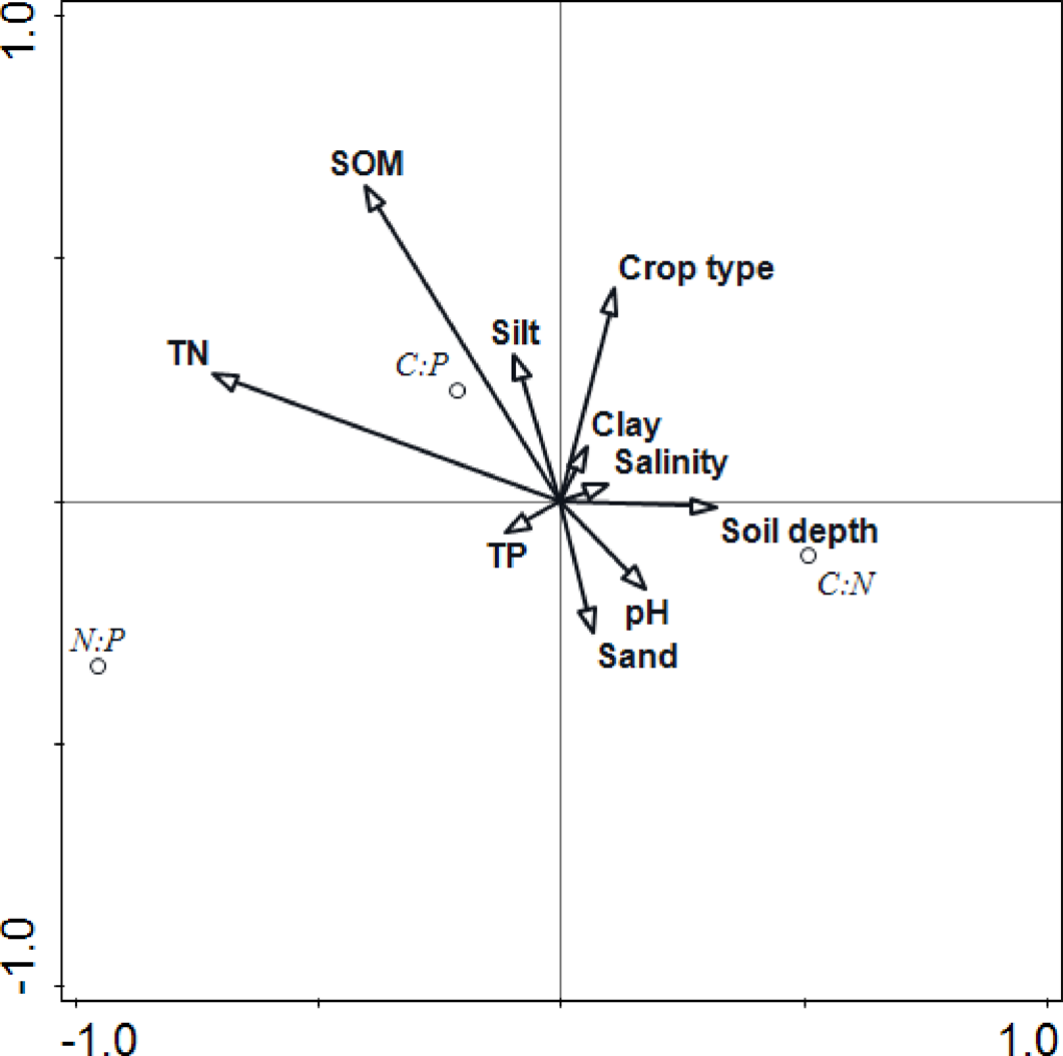
Canonical correlations between environmental factors and the soil C:N:P ratio.

Changes in soil physical and chemical properties are the major factors influencing differences in the C: N:P ratio. The redundant analysis revealed that the contributions of SOM, TN, TP and silt reached 82.8% (Fig. 3). SOM was positively correlated with the C: N, C: P, and N: P ratios (Fig. 2). For C: P, the difference reached a significant level throughout the entire soil depth profile (*P <* 0.05), whereas for N: P, it reached a significant level at soil depths of 0–20 cm, 20–40 cm, and 60–80 cm (*P <* 0.05). SOC is the main component of SOM. The SOM contains aliphatic C-H, polysaccharide, aromatic C = C and other functional groups, and is one of the main substances that provide C, N and P for soil, which is beneficial for soil to continuously provide nutrients for plants and serve as a buffer, driving basic biogeochemical processes, so it can also be used as an important index to evaluate soil fertility^31,32^. The mineralization of SOM can affect soil N and P mineralization and release N and P, which are wrapped in SOM particles^22^. The soil pH was significantly negatively correlated with the C: P and N: P ratios at the 0–20 cm soil depth (*P <* 0.05). pH can directly affect the dissolution of soil N and P and affect N and P conversion by regulating soil microbial activity^33–36^. Silt is an important part of soil aggregates. On the one hand, soil aggregates affect SOC, TN and TP through adsorption and resolution. On the other hand, the greater the sand content is, the greater the loss of N and P nutrients^12,37^.

The variation in soil depth is a minor factor affecting the C: N:P ratio. The redundant analysis revealed that the contribution of soil depth was only 0.2% (Fig. 3). Generally, the soil C: N, C: P, and N: P ratios decreased with increasing soil depth.

## Conclusion

The contents of soil C, N, and P and their stoichiometric ratios in the Hetao irrigation area of the Yellow River were investigated. The soil was weakly alkaline throughout the entire soil depth profile. For SF and OL, SOM was classified as Grade IV or V, respectively, TN was classified as Grade Ⅲ or V, respectively and TP was classified as Grade II in both SF and OL. Soil pH and salinity clearly increased, and OL shifted to SF. SOM, TN, and TP decreased significantly during conversion, indicating the occurrence of a soil degradation process. Moreover, the soil C:N, C:P, and N:P ratios were obviously lower than the national averages in China. With the transition from OL to SF, there was little difference in the C:N ratio, whereas the C: P and N: P ratios decreased significantly. Additionally, the soil C:N, C:P, and N:P ratios decreased overall with increasing soil depth.

In conclusion, soil degradation occurred during the crop type conversion process. The soil P mineralization rates increased, and the N limit improved with the shift from the OL to the SF. This study proposes that a strategic reduction in sunflower cultivation in the Hetao irrigation district can effectively alleviate soil salinization, mitigate soil degradation, and promote a steady increase in soil quality. The measures implemented demonstrate the potential to establish sustainable agricultural practices in this ecologically vulnerable region.

## Data Availability

The datasets generated during and/or analysed during the current study are available from the corresponding author on reasonable request.
